# Proteomic and Metabolomic Analyses Provide Insights into the Mechanism on Arginine Metabolism Regulated by tRNA Modification Enzymes GidA and MnmE of *Streptococcus suis*

**DOI:** 10.3389/fcimb.2020.597408

**Published:** 2020-12-11

**Authors:** Ting Gao, Fangyan Yuan, Zewen Liu, Wei Liu, Danna Zhou, Keli Yang, Rui Guo, Wan Liang, Geng Zou, Rui Zhou, Yongxiang Tian

**Affiliations:** ^1^ Key Laboratory of Prevention and Control Agents for Animal Bacteriosis, Ministry of Agriculture and Rural Affairs, Hubei Provincial Key Laboratory of Animal Pathogenic Microbiology, Institute of Animal Husbandry and Veterinary, Hubei Academy of Agricultural Sciences, Wuhan, China; ^2^ State Key Laboratory of Agricultural Microbiology, College of Veterinary Medicine, Huazhong Agricultural University, Wuhan, China; ^3^ Cooperative Innovation Center of Sustainable Pig Production, Wuhan, China

**Keywords:** *Streptococcus suis*, GidA-MnmE tRNA modification pathway, TMT, arginine metabolism, ADS, growth, pathogenicity

## Abstract

GidA and MnmE, two important tRNA modification enzymes, are contributed to the addition of the carboxymethylaminomethyl (cmnm) group onto wobble uridine of tRNA. GidA-MnmE modification pathway is evolutionarily conserved among Bacteria and Eukarya, which is crucial in efficient and accurate protein translation. However, its function remains poorly elucidated in zoonotic *Streptococcus suis* (SS). Here, a *gidA* and *mnmE* double knock-out (DKO) strain was constructed to systematically decode regulatory characteristics of GidA-MnmE pathway *via* proteomic. TMT labelled proteomics analysis identified that many proteins associated with cell divison and growth, fatty acid biosynthesis, virulence, especially arginine deiminase system (ADS) responsible for arginine metabolism were down-regulated in DKO mutant compared with the wild-type (WT) SC19. Accordingly, phenotypic experiments showed that the DKO strain displayed decreased in arginine consumption and ammonia production, deficient growth, and attenuated pathogenicity. Moreover, targeted metabolomic analysis identified that arginine was accumulated in DKO mutant as well. Therefore, these data provide molecular mechanisms for GidA-MnmE modification pathway in regulation of arginine metabolism, cell growth and pathogenicity of SS. Through proteomic and metabolomic analysis, we have identified arginine metabolism that is the links between a framework of protein level and the metabolic level of GidA-MnmE modification pathway perturbation.

## Introduction

tRNA modifications are widely distributed in Bacteria and Eukarya, some of which are conserved in nature. Cellular health and growth requires protein synthesis to be both efficient and accurate, in order to avoid producing defective or unstable proteins (Manickam et al., 2016). Until now, more than 90 modified nucleosides have been found in tRNA (Hori, 2014), such as base isomerization, thiolation, deamination, methylation of a ribose or base, or complex hypermodifications and so on (Boccaletto et al., 2018). Many modifications inside the structural core of the tRNA are essential to stabilizing the overall structure of the tRNA, thus, loss of these modifications can lead to rapid degradation of hypomodified tRNAs (Yu et al., 2019). In bacteria, modified nucleosides of different chemical structures, present in different positions, and in different species of the tRNA all prevent frameshifts errors (Urbonavicius et al., 2001; Schweizer et al., 2017). Therefore, tRNA modifications are essential for the control of bacterial gene expression under stressful and changing environments (Gustilo et al., 2008). To date, hundreds of tRNA modification enzymes have been identified and there are great diversity of enzymes in wobble position modification (Shippy and Fadl, 2014; Hou et al., 2015).

GidA and MnmE, two important tRNA modification enzymes, are involved in the addition of the cmnm group in the position 5 of the wobble uridine 34 of tRNAs that read codons ending with A or G (Yim et al., 2006; Shippy and Fadl, 2015; Gao et al., 2019). Correspondingly, the orthologs of GidA and MnmE are called MTO1 and MSS1/GTPBP3 in yeast and human respectively (Zhu et al., 2015). There are three main structural or functional roles for this modification: (i) proper codon–anticodon interaction at the decoding center of the ribosome (Fislage et al., 2014), (ii) appropriate decoding of mRNA (Urbonavicius et al., 2001), (iii) maintaining the translational reading frame (Tükenmez et al., 2015). Lack of GidA-MnmE tRNA modification pathway lead to pleiotropic effect, affecting diverse phenotypic traits. This may result from a reduced efficiency of wobble uridine to read the third base of codons, being forced by GidA and MnmE throughout modification of specific tRNAs. Thus, GidA-MnmE pathway has been implicated to be a major regulatory mechanism on pathogenicity (Shippy and Fadl, 2014). Studies have been reported that several bacterial pathogens attenuated in virulence as deletion of GidA-MnmE pathway: *Salmonella enterica* Serovar Typhimurium, *Streptococcus mutans*, *Pseudomonas syringae*, *Aeromonas hydrophila*, for example. While, in yeast, MTO1 and MSS1 which are the homologues of GidA and MnmE localize in mitochondria, and their mutants are associated with respiratory defects. Moreover, human disease, hypertrophic cardiomyopathy has been related to defects in this tRNA modification pathway (Zhu et al., 2015; Boutoual et al., 2018; Finsterer and Zarrouk-Mahjoub, 2018). Taken together, the above studies stress the importance of this conserved tRNA modification pathway in the cellular process. However, at present, little is known about the GidA-MnmE pathway in the zoonotic pathogen *Streptococcus suis*.

*Streptococcus suis* is an important zoonotic pathogen that is responsible for severe economic losses in the pig industry and poses a significant threat to human health (Lun et al., 2007; Feng et al., 2014; Goyette-Desjardins et al., 2014; Lin et al., 2019). This bacterium can cause a variety of diseases, including meningitis, arthritis, septicemia, pneumonia, endocarditis, and toxic shock-like syndrome. After two epidemics outbreak in China with high mortality that occurred in 1998 and 2005 (Dutkiewicz et al., 2017) (Tang et al., 2006; Lun et al., 2007; Ye et al., 2009), as well as the disclosure of the high morbidity in other East Asia countries, human and swine infections caused by SS have become a hot research field. Based on the capsular polysaccharides, 33 serotypes have been identified in the past decades. Among these serotypes, *Streptococcus suis* serotype 2 (SS2) is reported to be the most virulent and prevalent strain commonly associated with diseases in pigs and human beings (Hughes et al., 2009; Okura et al., 2016). Numerous virulence factors are responsible for the pathogenecity of SS2, such as capsular polysaccharide (Cps), muramidasereleased protein, suilysin (Sly), extracellular factor, fibrinonectin and fibrinogen-binding proteins (FbpS), enolase, arginine deiminase system (ADS), glyceraldehyde-3-phosphate dehydrogenase (GAPDH), inosine 5-monophosphate dehydrogenase (IMPDH), and so on (Segura et al., 2017; Dutkiewicz et al., 2018; Lin et al., 2019; Xia et al., 2019). There are four stages for SS to successfully survive in the host: the first stage is to adherence to and colonization of mucosal of the upper respiratory tract; subsequently, the second stage is translocation across epithelial cell barriers; then, the third stage is to reach the bloodstream and disseminate through the circulatory system; and finally invades different organs of the host (Fittipaldi et al., 2012; Dutkiewicz et al., 2018). In all stages of the pathogenic process, SS may also encounter adverse situations, thus, SS must adapt metabolically to survive *in vivo* and maintain pathogenesis (Willenborg et al., 2016). As the upstream process of metabolism, many proteins are regulated (up or down) at the translation level in response to environmental stimuli and surroundings change. However, the precise mechanisms of preferential regulation of proteins by SS during specific stages of host infection remains elusive.

To systematacially understand the regulatory mechanism of GidA-MnmE pathway in SS2, we constructed a *gidA*-*mnmE* DKO mutant of SS2, and investigated its biological characterizations using “-omics” approaches. By using the TMT-labelled proteomic approach, we identified differentially expressed proteins (DEPs) between the *gidA*-*mnmE* mutant strain and the WT strain SC19. The targeted metabolomics was performed to investigate the distinctions of amino acid metabolites between *gidA*-*mnmE* mutantand WT using the multiple reaction monitoring (MRM) mode of a LC-MS/MS. The analyses of both proteomics and metabolomics provide functional context that GidA-MnmE pathway can regulate arginine metabolism, cell growth, as well as pathogenicity of SS2.

## Materials and Methods

### Ethics Statement

All mice used in this study were purchased from the Wuhan Institute of Biological Products (Wuhan, China). All experiments with the SS2 were performed in biosafety cabinets in biosecurity level 3 laboratory. All animal studies were conducted in strict accordance with the animal welfare guidelines of the World Organization for Animal Health. The animal study protocol was approved by the Ethics Committee of Institute of Animal Husbandry and Veterinary, Hubei Academy of Agricultural Sciences (Wuhan, China) and conducted in accordance with the Hubei Province Laboratory Animal Management Regulations of 2005. All efforts were made to minimize animal suffering.

### Bacterial Strains, Plasmids, and Culture Media

The bacterial strains and plasmids used in this study are listed in **Table 1**, strain SC19 was isolated from a diseased pig during an epidemic outbreak in 2005 in Sichuan, China (Li et al., 2009). The *mnmE* deletion mutant of SC19 (Δ*mnmE*) obtained from our previous research were also used in this study (Gao et al., 2019). The SS2 were grown in Todd-Hewitt broth (THB; Oxoid, Basingstoke, England) or on THB agar (THA; Oxoid, Basingstoke, England) plates supplemented with 5% sheep blood (Maojie, Nanjing, China) at 37°C. The arginine metabolic pathway study was performed using a chemically defined medium (van de Rijn and Kessler, 1980). *Escherichia coli* (*E. coli*) strain DH5α (Vazyme, Nanjing, China) used as host strain for cloning, were grown in LB broth (Difco Laboratories, Franklin Lakes, NJ, USA) or on LB agar plates at 37°C. If necessary, erythromycin (90 μg/ml), spectinomycin (100 μg/ml), and streptomycin (20 μg/ml) were supplemented to promote bacterial selection.

**Table 1:** Bacterial strains and plasmid used in this study.

Strain or plasmid	Characteristics and function^a^	Source or reference
Bacterial strains		
SC19	*S. suis* serotype 2, the wide- type (Strep^r^)	(Li et al., 2009)
* ΔmnmE*	SC19 *mnmE*::*erm* (Strep^r^ Erm^r^)	In this lab
* ΔgidAΔmnmE*	SC19 *gidA mnmE*::*erm* (Strep^r^ Erm^r^)	This study
* E. coli* DH5α	Cloning host for recombinant vector	Vazyme
Plasmids		
pSET4s	*E. coli* - *S. suis* Shuttle vector; Spc^r^	(Takamatsu et al., 2001)
pSET4s-G	Derived from pSET4s for knocking out gene *gidA* in *ΔmnmE*; Spc^r^ Erm^r^	In this lab

### Construction and Confirmation of *gidA*-*mnmE* DKO Mutant (Δ*gidA*Δ*mnmE*)

The Δ*gidA*Δ*mnmE* was obtained using an homologous recombination method. Primers used in this study were designed according to the genome sequence of SS2 strain 05ZYH33 (GenBank accession number: CP000407) and are listed in **Table 2**. The thermosensitive suicide vectors for *gidA* knockout pSET4s-G (Gao et al., 2016) was electroporated into Δ*mnmE*. Then the Δ*gidA*Δ*mnmE* strain was screened on THB plates using PCR. To confirm the DKO strain, the *gidA* gene and *mnmE* gene were amplified by PCR using the primer pairs *gidA*-F/*gidA*-R and *mnmE*-F/*mnmE*-R, and the primer pairs *cps2J*-F/*cps2J*-R were used to amplify the specific gene *cps2J* of SC19. Moreover, Primers GT-F/GT-R and MT-F/MT-R were used to amplify the upstream to downstream regions of *gidA* and *mnmE* in SC19 and DKO strain, respectively, and the resulting DNA fragments were confirmed by DNA sequencing.

**Table 2:** Primers used for PCR amplification and detection.

Primers	Primers sequence (5′—3′)	Amplification for
*gidA*-F	CGGGATCCATGACACACACATTTGCAGA	*gidA* gene
*gidA*-R	CGCTCGAGTTAGTGACTGTCCTTTGATTT	
*mnmE*-F	CGGGATCCATGACACACACATTTGCAGA	*mnmE* gene
*mnmE*-R	CGCTCGAGTTAGTGACTGTCCTTTGATTT	
GT-F	GCTTTTGTGGACTTAGAGAAATTGG	Upstream to downstream regions of *gidA*
GT-R	ACGAACCTCAGCCATATTGAGATTT	
MT-F	GACAAAATAGCTGGTGTAATAAAG	Upstream to downstream regions of *mnmE*
MT-R	CTGTGTATGAAGGAGTTGAGGC	
*cps2J*-F	TGATAGTGATTTGTCGGGAGGG	*cps2J* gene
*cps2J*-R	GAGTATCTAAAGAATGCCTATTG	
*arcA*-F	ATGAGAGGCAGGAAAGCGTACGGTC	*arcA* gene
*arcA*-R	AAGGCATCATGCTCTTTCTGTG	
*arcB*-F	TGTTGGCGGACTACTTGACTG	*arcB* gene
*arcB*-R	ACGTGCTCCACTTTCTTTCG	
*arcC*-F	ACCCATCGGCTAAAGCACAA	*arcC* gene
*arcC*-R	TTCCGAATCAGCAGCAAGGT	
16S RNA-F	GTAGTCCACGCCGTAAACG	16S RNA
16SRNA-R	TAAACCACATGCTCCACCGC	

To further confirm the mutant strain Δ*gidA*Δ*mnmE*, we also performed RT-PCR (Tan et al., 2015). Briefly, RNA was isolated using the Bacterial RNA Kit (Omega Bio-Tek, Inc., Norcross, GA, USA) in accordance with the manufacturer’s instructions. In addition, cDNA was synthesized using the HiScript R II Q Select RT SuperMix for qPCR kit (Vazyme, Nanjing, China) in accordance with the manufacturer’s instructions. For RT-PCR, primers *gidA*-F/*gidA*-R and and *mnmE*-F/*mnmE*-R listed in **Table 2** were used to confirm the deletion of *gidA* and *mnmE* gene, respectively. To exclude single nucleotide polymorphisms (SNPs) effect, we also performed whole genome sequencing of mutant strain Δ*gidA*Δ*mnmE*.

### TMT Labeled Proteomics

#### Protein Extraction, Digestion, and Labeling

SC19 and Δ*gidA*Δ*mnmE* cells at mid-log phase were cultured in THB as described above. Three independent biological replicates of bacterial pellets were then treated with SDT buffer (4% SDS, 100 mM Tris-HCl, 1 mM DTT, pH 7.6) and heated for 15 min at 100°C. Proteins were extracted in SDT buffer as previously described (Wiśniewski et al., 2009), and protein concentrations in the supernatants were determined through Bradford protein assay. Each sample (100 µg) were digested with 3 µg of trypsin (Sigma-Aldrich Corporation) at 37°C for 16 h. Pierce high pH reversed-phase fractionation kit (Thermo Fisher Scientific, MA, USA) was used to fractionate TMT-labeled digest samples into 15 fractions by an increasing acetonitrile step-gradient elution according to instructions. The resulting tryptic peptides were labeled according to the protocol of TMT Reagent Kit (Thermo Fisher Scientific, MA, USA).

#### LC-MS/MS Analysis

Each fraction was injected for nanoLC-MS/MS analysis. The peptide mixture was loaded onto a reverse phase trap column (Thermo Scientific Acclaim PepMap100, 100 µm × 2 cm, nanoViper C18) connected to the C18-reversed phase analytical column (Thermo Scientific Easy Column, 10 cm long, 75 μm inner diameter, 3 μm resin) in buffer A (0.1% Formic acid) and separated with a linear gradient of buffer B (84% acetonitrile and 0.1% Formic acid) at a flow rate of 300 nl/min controlled by IntelliFlow technology. The mass spectrometer was operated in positive ion mode. MS data was acquired using a data-dependent top10 method dynamically choosing the most abundant precursor ions from the survey scan (300–1,800 m/z) for HCD fragmentation. Automatic gain control (AGC) target was set to 1e6, and maximum inject time to 50 ms. Dynamic exclusion duration was 60.0 s. Survey scans were acquired at a resolution of 70,000 at m/z 200 and resolution for HCD spectra was set to 35,000 at m/z 200 (TMT 10plex), and isolation width was 2 m/z. Normalized collision energy was 30 eV and the underfill ratio, which specifies the minimum percentage of the target value likely to be reached at maximum fill time, was defined as 0.1%.

#### Data Analysis

MS/MS spectra were searched using MASCOT engine (Matrix Science, London, UK; version 2.2) against 83,725 S. suis protein-coding sequences deposited in the Uniprot database and embedded into Proteome Discoverer 1.4. The search was conducted with trypsin applied as a specific enzyme and parameters used for normal peptides as follows: peptide mass tolerance, 20 ppm; fragment mass tolerance, 0.1 Da; max missed cleavages, 2; fixed modifications, carbamidomethyl (C), TMT 10plex (N-term), TMT 10plex (K); variable modifications: oxidation (M), TMT 10plex (Y); database pattern, decoy; and false-discovery rate ≤0.01. Each of the identified proteins involved at least one unique peptide. Protein quantification was accomplished by correlating the relative intensities of reporter ions extracted from tandem mass spectra to that of the peptides selected for MS/MS fragmentation. Fold change (Δ*gidA*Δ*mnmE*/SC19) > 1.2 and < 0.83, and a *p*-value of <0.05 were used to represent up- or down-regulation, respectively. The mass spectrometry proteomics data have been deposited to the ProteomeXchange Consortium *via* the PRIDE partner repository with the dataset identifier PXD012716.

### Determination of Arginine Deiminase (AD/ArcA) Activity

AD activity was determined according to the protocol of Degnan (Degnan et al., 1998) as described previously (Winterhoff et al., 2002). The conversion of L-arginine to L-citrulline was catalyzed by arginine deiminase. Briefly, bacteria were grown in CDM medium and harvested by centrifugation. Then, the bacterial pellets were lysed through ultrasonication. The resulting lysates were incubated for 2 h in 0.1 M potassium phosphate buffer containing 10 mM L-arginine at 37°C. The enzymatic reaction was stopped by adding 250 μl of a 1:3 (v/v) mixture of 95% H_2_SO_4_ and 85% H_3_PO_4_, and 250 μl of 3% diacetyl monooxime solution were added to the samples incubating for 15 min at 100°C. Production of citrulline was determined colorimetrically at an OD450 nm and results are given in nmol citrulline produced in 1 h per mg whole cell protein.

### Determination of Ammonia in the Culture Supernatant

Ammonia in the culture supernatant of WT and DKO mutant was performed using an ammonia colorimetric assay kit (Sigma-Aldrich, MO, USA) in accordance with the manufacturer’s instructions.

### RT-qPCR to Detect the arcABC Genes at mRNA Level

RT-qPCR was performed to examine the mRNA levels of the *arcABC* genes described above. Primers *arcA*-F/*arcA*-R, *arcB*-F/*arcB*-R, *arcC*-F/*arcC*-R, and 16sRNA listed in **Table 2** were used to determine *arcABC* genes expression at mRNA Level.

### Detection of Growth Curves and Colony Size

The growth rates of WT and *gidA*-*mnmE* DKO mutant over a period of 10 h were determined through the measurement of the density changes represented by optical density 600 nm (OD600 nm) values and colony-forming unit (CFU) counts of the cultures. OD600 nm values of different strains were read every hour, meanwhile, strain samples were plated (100 μl) of various dilutions of the cultures on THA plates. Overnight incubation of the plates at 37°C, the number of CFU per milliliter of sample was calculated. Single bacterial colony size of WT and *gidA*-*mnmE* DKO mutant at the same incubation period were also monitored.

### Transmission Electron Microscopy Analysis of the Bacteria

To get an overview of the cell size of SC19 and DKO mutant, TEM was carried out as following steps. 20 μl of bacterial suspensions were dropped onto the copper grid with carbon film for 3-5min, the samples were then stained with 2% phosphotungstic acid for 2min and dried at room temperature. Cell morphology was observed using an HT-7700 TEM ((HITACHI, Ltd., Tokyo, Japan). 6 bacterial cells were randomly chosen from the images to measure the cell size of SC19 and DKO mutant, and then data was statistically analyzed on GraphPad prism 5.

### Pathogenicity Assay in Mice

To detect the virulence of GidA-MnmE pathway in *S. suis*, a total of 30 female 6-week-old specific-pathogen-free (SPF) Kun-Ming mice (10 mice per group) were intraperitoneally infected with 3 × 10^9^ CFU/mouse of either SC19 or Δ*gidA*Δ*mnmE*. Physiological saline was applied as a negative control. Then the morbidity, mortality, and clinical symptoms of all the mice were observed for 7 days.

To evaluate the eﬀect of GidA and MnmE on colonization in blood and different organs, competitive colonization experiment of SC19 and Δ*gidA*Δ*mnmE* was performed as previously. A total of 15 SPF Kun-Ming mice (6-week-old) were co-infected with SC19 and Δ*gidA*Δ*mnmE* at the ratio 1:1 (1 × 10^8^ CFU/mouse). Physiological saline was applied as negative control in five mice. Bacterial counts in blood, brain, and lung were collected at 12 h, 1 day, and 3 days post infection (dpi). The samples were homogenized after weighing, and serial dilutions were plated onto TSA agar with 20 µg/ml streptomycin (Strep) and 90 µg/ml erythromycin (Erm). The number of live bacteria on TSA agar plates with Erm or Strep was calculated as Δ*gidA*Δ*mnmE*, sum of SC19 and Δ*gidA*Δ*mnmE*, while, Erm was calculated as sum of SC19 and Δ*gidA*Δ*mnmE*, respectively. The number of SC19 was calculated as TSA^Strep^ -TSA^Erm^.

### Amino Acid Metabolite Quantification and Metabolomics

#### Sample preparation

Bacterial pellets were resuspended in a 1.5 ml centrifuge tubes after addition of 1 ml ice-cold methanol/acetonitrile/water (2:2:1, v/v/v), then mixed by vortex. Protein deposition was induced by ultrasound for 30 min in the ice bath. The homogenate were then incubation at -20°C for 1 h. After that, the samples were centrifuged at 14,000 rcf for 20 min at 4°C. The supernatant were transferred and treated with vacuum drying evaporated. For the LC–MS/MS analysis, the samples were re-dissolved in 100 μl acetonitrile/water (1:1, v/v) solvent. To monitor the stability and repeatability of instrument analysis, quality control (QC) samples were prepared by pooling 10 μl of each sample and these were analyzed together with the other samples.

#### LC–MS/MS Conditions

The samples were separated using an auto-sampler Agilent 1290 HPLC system (Agilent Technologies, CA, USA). The mobile phase consisted of 0.1% formic acid in 25 mM ammonium formate (A) and 0.1% formic acid in acetonitrile (B). 2 μl aliquot of each sample was automatically injected onto the column at 4°C with a column temperature of 40°C and a flow rate of 300 μl/min. The elution gradient was as follows: 0-12 min, liquid B changes linearly from 90% to 70%; 12–18 min, liquid B changes linearly from 70% to 50%; 18–25 min, liquid B changes linearly from 50% to 40%; 30–30.1 min, liquid B changes linearly from 40% to 90%; 30.1–37 min, liquid B is maintained at 90%.

The 5500 QTRAP mass spectrometer (AB SCIEX), was used for mass spectrometry analysis in positive ion mode. Electrospray ionization (ESI) source conditions were set as following: source temperature 500°C; ion source gas1 (Gas1): 40; ion source gas2 (Gas2): 40; curtain gas (CUR): 30; ion Spray Voltage Floating (ISVF): 5000 V. MRM mode was used to detect amino acids. Fold change (Δ*gidA*Δ*mnmE*/SC19) > 2 and < 0.5, and a *p*-value of <0.05 were used to represent up- or down-regulation, respectively.

#### Data analysis

MultiQuant software was used to extract peak area and retention time of the chromatogram. Metabolites were identified by modified retention time and by comparison to the standards of amino acids and its derivatives.

### Statistical Analysis

Unless otherwise specified, statistical analyses were performed *via* unpaired Student’s t-tests in GraphPad Prism 5 software (GraphPad Software, CA, USA). All data are expressed as the mean ± the standard error of the mean (SEM) and all experiments were performed in triplicate at least three times. A *p*-value of less than 0.05 was considered to be statistically significant.

## Results

### TMT Quantitative Proteomic Analysis of the DEPs Obtained from Δ*gidA*Δ*mnmE* and SC19

As GidA and MnmE were important tRNA modification enzyme contributed in efficient and accurate protein translation, we performed proteomics to systematically analyze the function of GidA and MnmE modification pathway in SS2.

**Figure 1:**
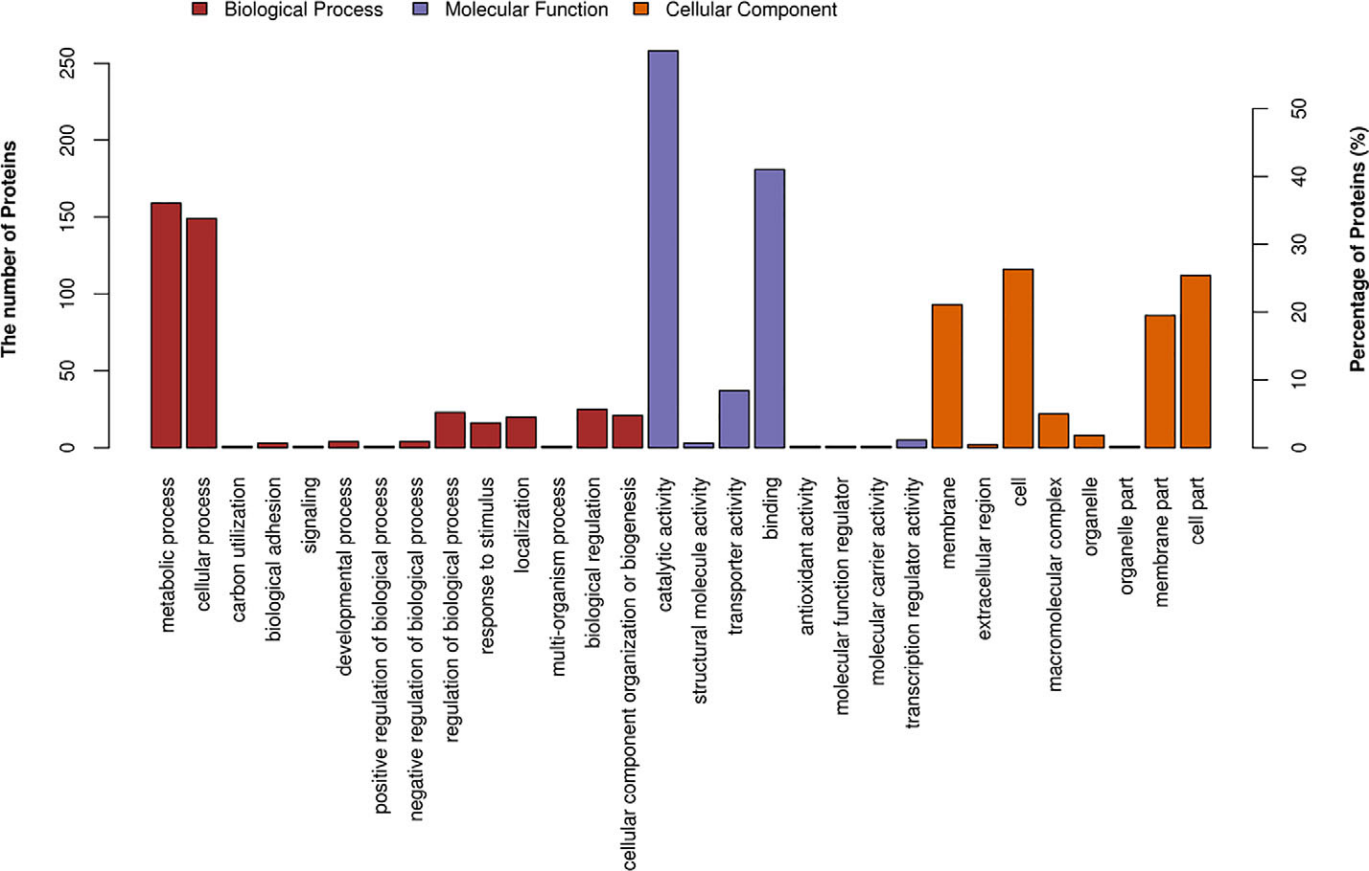
Classification of differentially expressed proteins in Δ*gidA*Δ*mnmE* according to gene ontology (GO) annotation on three ontologies (biological process, molecular function, and cellular component).

To get a glimpse of proteomic dysregulation, protein expression profiles were determined using TMT-based quantitative proteomics for Δ*gidA*Δ*mnmE* and SC19. A total of 1,619 proteins were detected and quantified, among which 441 were differentially expressed (27% of expressed proteins) in Δ*gidA*Δ*mnmE* compared to SC19, with 218 up-regulated and 223 down-regulated (*p* < 0.05, fold change > 1.2). According to gene ontology (GO) and kyoto encyclopedia of genes and genomes (KEGG) analysis, the main function of the 441 DEPs were involved in catalytic activity, binding, transporter activity, transcription regulator activity, and structural molecule activity. The DEPs were also taking part in several biological process, such as metabolic process, cellular process, biological regulation, regulation of biological process, and cellular component organization or biogenesis (**Figure 1**), which are the most important process for SS in response to stimulus and adaption to environmental change.

### Functional Enrichment Analysis

To further explore the impact of DEPs in cell physiological process and discover internal relations between DEPs, enrichment analysis was performed. GO enrichment on three ontologies (biological process, molecular function, and cellular component) enrichment analyses were applied based on the Fisher’ exact test (p < 0.05), considering the whole quantified protein annotations as background dataset. 74 GO terms were significantly perturbed by the deletion of *gidA* and *mnmE*. Among these, organic acid biosynthetic process, carboxylic acid biosynthetic process, coenzyme biosynthetic process, fatty acid biosynthetic process, fatty acid metabolic process, cellular amino acid biosynthetic process, as well as arginine metabolic process, and so on (**Table 3**), are crucial for bacterial cell growth, environmental adaption and virulence. Arginine metabolic process is a primary point of our concern.

**Table 3:** Gene ontology (GO) enrichment on ontologies for biological process, molecular function, and cellular component.

GO ID	Term	Category	Test protein	Reference protein	*p*-value	Richfactor
**Acid biosynthesis and metabolism**						
GO:0046394	carboxylic acid biosynthetic process	biological process	32	69	0.0003	0.4637
GO:0016053	organic acid biosynthetic process	biological process	32	72	0.0009	0.4444
GO:0043648	dicarboxylic acid metabolic process	biological process	11	22	0.0185	0.5000
GO:0043650	dicarboxylic acid biosynthetic process	biological process	9	18	0.0325	0.5000
GO:0072330	monocarboxylic acid biosynthetic process	biological process	7	12	0.0225	0.5833
GO:0046943	carboxylic acid transmembrane transporter activity	molecular function	6	11	0.0503	0.5454
GO:0005342	organic acid transmembrane transporter activity	molecular function	6	11	0.0503	0.5454
**Fatty acid biosynthesis and metabolism**						
GO:0006633	fatty acid biosynthetic process	biological process	6	10	0.0295	0.6000
GO:0006631	fatty acid metabolic process	biological process	6	10	0.0295	0.6000
**Amino acid biosynthesis and metabolism**						
GO:0008652	cellular amino acid biosynthetic process	biological process	21	53	0.0316	0.3962
GO:0006525	arginine metabolic process	biological process	5	8	0.0386	0.6250
GO:0006551	leucine metabolic process	biological process	3	3	0.0201	1
GO:0009098	leucine biosynthetic process	biological process	3	3	0.0201	1
**Purine biosynthesis and metabolism**						
GO:0009113	purine nucleobase biosynthetic process	biological process	3	3	0.0201	1
GO:0006144	purine nucleobase metabolic process	biological process	4	5	0.0213	0.8000
GO:0009127	purine nucleoside monophosphate biosynthetic process	biological process	10	20	0.0245	0.5000
GO:0009168	purine ribonucleoside monophosphate biosynthetic process	biological process	10	20	0.0245	0.5000
GO:0042278	purine nucleoside metabolic process	biological process	6	10	0.0295	0.6000

### Growth and Division-Associated Proteins

Inactivating GidA-MnmE modification pathway affects the growth rate of SS2, about 15 DEPs involved in cell growth and division were found to be down-regulated in the DKO strain (**Table 4**). Among these DEPs, 8 were involved in DNA replication, recombination, and repair (XerD, YjqA, Rnr, Ribonuclease BN, RnhC, DnaE, MutS, TopA); 2 were involved in protein synthesis and posttranslational control (IF-2, PrpC); 3 were involved in cell wall biosynthesis (MurD, MurG, Pbp1A); and 2 were involved in cell division (StpK, FtsK).

**Table 4:** Differentially expressed proteins associated with cell growth and division, virulence, and fatty acid biosynthesis between the △*gidA*△*mnmE* and SC19.

Protein name	Locus	Functions	Ratio(△gidA△*mnmE*/SC19)	Uniquepeptides	Sequence coverage(%)
**CELL GROWTH AND DIVISION**					
IF-2	SSU05_0272	Translation initiation factor 2	0.5757	16	47.28
Pbp1A	SSU05_0414	Penicillin-binding protein	0.8227	24	36.88
StpK	SSU05_0428	Serine/threonine protein kinase	0.7411	20	35.54
RnBN		Ribonuclease BN	0.8239	2	5.54
RnhC	SSU05_0226	Ribonuclease HIII	0.6607	3	6.76
PrpC	SSU05_0472	Protein phosphatase	0.7162	11	59.59
MurD	SSU05_0476	D-glutamic acid-adding enzyme	0.7614	19	55.68
MurG	SSU05_0477	UDP-N-acetylglucosamine	0.7703	7	28.81
DnaE	SSU05_0542	DNA-directed DNA polymerase	0.6931	20	20.66
TopA	SSU05_0985	DNA topoisomerase I	0.7542	23	35.29
FtsK	SSU05_1335	DNA segregation ATPase	0.7902	23	27.50
Rnr	SSU05_1391	Ribonuclease R	0.7188	1	33.08
YjqA	SSU05_1576	Superfamily I DNA/RNA helicase	0.7017	4	27.56
XerD	SSU05_1702	Site-specific tyrosine recombinase XerD-like protein	0.6141	2	7.82
MutS	SSU05_2123	DNA mismatch repair protein	0.8212	17	22.10
**VIRULENCE-ASSOCIATED PROTEINS**					
SadP	SSU05_0272	Translation initiation factor 2 GTPase	0.5757	16	47.28
ArcA	SSU05_0624	Arginine deiminase	0.4513	21	56.48
ArcB	SSU05_0626	Ornithine carbamoyltransferase	0.5403	13	45.99
ArcC	SSU05_0627	Carbamate kinase	0.3812	2	6.67
ArgR	SSU05_0631	Arginine repressor	1.2236	4	26.71
Sao	SSU05_1371	Surface antigen	0.6282	28	64.33
HlyC	SSU05_1668	Hemolysin C	0.5836	1	3.60
FbpS	SSU05_1942	Fibronectin/fibrinogen binding protein	0.8209	15	30.07
ZnuA	SSU05_2086	High-affinity zinc uptake system protein	0.7775	1	51.14
IMPDH	SSU05_2183	Inosine 5’-monophosphate dehydrogenase	0.6458	3	83.16
**FATTY ACID BIOSYNTHESIS**					
AccA	SSU05_1796	Acetyl-coenzyme A carboxylase carboxyl transferase subunit alpha	0.3256	6	19.46
AccD	SSU05_1797	Acetyl-coenzyme A carboxylase carboxyl transferase subunit beta	0.2778	8	31.25
AccC	SSU05_1799	Biotin carboxylase	0.3785	10	28.88
FabZ	SSU05_1800	3-hydroxyacyl-[acyl-carrier-protein] dehydratase	0.3905	2	17.14
AccB	SSU05_1801	Biotin carboxyl carrier protein of acetyl-CoA carboxylase	0.4501	1	8.16
FabF	SSU05_1802	3-oxoacyl-[acyl-carrier-protein] synthase 2	0.2011	8	26.52
FabG1	SSU05_1803	3-ketoacyl-ACP reductase	0.2392	13	80.33
FabD	SSU05_1804	Malonyl CoA-acyl carrier protein transacylase	0.4979	6	23.86
FabI	SSU05_1805	2-nitropropane dioxygenase	0.2211	2	39.56
AcpP	SSU05_1806	Acyl carrier protein	0.5513	1	14.86
FadB	SSU05_1809	Enoyl-CoA hydratase	0.4717	9	34.22

### Arginine Metabolism and Virulence-Associated Proteins

About 10 DEPs associated with arginine metabolism and virulence were found to be down-regulated in the DKO strain (**Table 4**). These DEPs were classified as three parts: 4 were involved in surface/secreted components (Sao, HlyC, FbpS, ZnuA); 5 were involved in enzyme complexes (SadP, ArcA, ArcB, ArcC, IMPDH); 1 was involved in transcriptional regulators (ArgR). Among these 10 DEPs, 4 were involved in arginine metabolism (ArcA, ArcB, ArcC, ArgR).

### Fatty Acid Biosynthesis-Associated Proteins

Fatty acid biosynthesis plays a vital role in the pathogenicity of several bacteria and adaption of survival conditions in the host (Ghazaei, 2018; Rameshwaram et al., 2018). The *gidA* and *mnmE* deletion resulted in down-regulation of 11 DEPs associated with fatty acid biosynthesis. Among these DEPs, FabF, FabI, and FabG1 were decreased 4 to 5 fold greater in Δ*gidA*Δ*mnmE* than that of SC19 (**Table 4**).

### GidA-MnmE Pathway Regulated AD Activity and Ammonia Production

**Figure 2:**
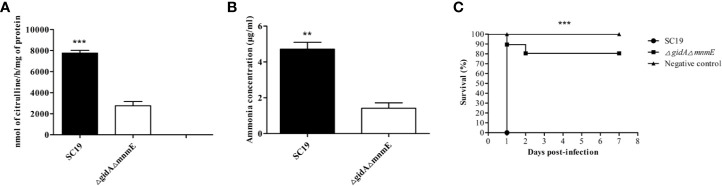
Regulation of AD Activity, ammonia production and pathogenicity by GidA-MnmE pathway. **(A)** AD activities of SC19 and Δ*gidA*Δ*mnmE*. Results were expressed as nanomoles of citrulline produced per hour per milligram of whole cell protein. **(B)** Ammonia production of SC19 and Δ*gidA*Δ*mnmE*. Ammonia production in supernatant of SC19 and Δ*gidA*Δ*mnmE* are given as μg ammonia/ml. Data are presented as the mean ± SEM of three independent experiments. Statistical significance was determined by two-tailed *t* test (**, *p* < 0.01; ***, *p* < 0.001). **(C)** Survival curves for mice in infection experiment. Ten mice in each group were separately injected intraperitoneally with 3 × 10^9^ CFU/mouse of SC19 or Δ*gidA*Δ*mnmE*. Ten mice were inoculated with saline and served as negative control. Significant difference in survival between different groups was analyzed by Log Rank test (*p* < 0.001).

The whole-cell protein extract from wild-type SC19 and DKO strain were performed to analyze AD activity. As a result, the AD activity was 7756 nmol of citrulline/h/mg of protein for SC19, while it was 2,756 nmol of citrulline/h/mg of protein for DKO strain (**Figure 2A**). These data demonstrate that the deletion of *gidA*-*mnmE* modification pathway down-regulated activities of ArcA. Therefore, we hypothesized that this tRNA modification pathway might affect SS2 ArcA expression through direct effects on translation or indirect regulatory effect.

To further investigate the regulatory role of GidA-MnmE pathway on arginine metabolism, we determined ammonia production of SC19 and DKO strain. The ammonia production for SC19 was 4.71 μg/ml, in contrast, the ammonia production for DKO strain was 1.18 μg/ml (**Figure 2B**), indicating that GidA-MnmE pathway is required for adequate function of the ADS of SS2.

Proteomic analysis identified core enzymes of arginine metabolism (ArcA, ArcB, ArcC) for Δ*gidA*Δ*mnmE* were down-regulated two to three fold than SC19, and this result was consistent with biochemical study presented above.

mRNA levels of the *arcA* was down-regulated to 2 fold in Δ*gidA*Δ*mnmE* than that in SC19, however, mRNA levels of the *arcB* and *arcC* were unchanged (**Figure S2**).

### GidA-MnmE Pathway Involves in the Growth Characterization of SC19

*gidA* and *mnmE* double-knock mutant was confirmed by PCR, RT-PCR (**Figure S1**) and whole genome sequencing. There were 2 SNPs in the DKO strain compared to the wild-type SC19, and the resulting 2 SNPs did not cause any changes in protein function.

**Figure 3:**
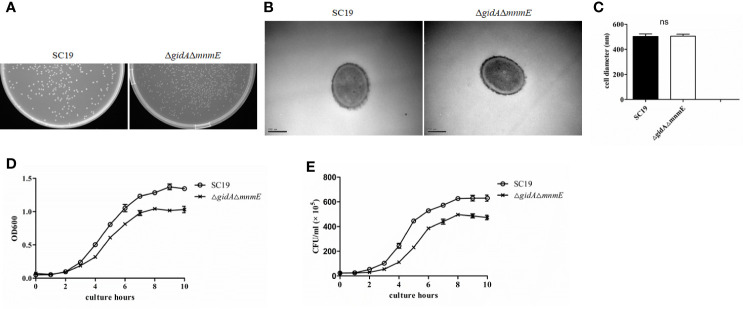
Micrographs and growth characterization of SC19 and Δ*gidA*Δ*mnmE*. **(A)** Colonies of SC19 and Δ*gidA*Δ*mnmE* cultured overnight on THA plates. **(B)** Transmission electron microscopy micrographs of the strains. **(C)** Quantification of transmission electron microscopy (TEM) analysis of cell size. **(D)** Growth curves of the strains. Bacterial cell density was measured spectrometrically at 600 nm. Data were collected at the indicated times. **(E)** Colony-forming unit (CFU) count of the strains. Separate aliquots of the bacterial suspensions were serially diluted and plated to determine CFU numbers per milliliter. Data were collected at the indicated times.

After obtaining the DKO mutant, we observed that the colonies of the mutant appeared smaller than those of SC19 when cultured on TSA plates overnight (**Figure 3A**). There are two reasons for this phenotype: (i) the smaller cellular size of the DKO mutant or (ii) reduced growth. Therefore, TEM and OD600 nm assay was conducted to figure out this phenomenon. However, there was no obvious difference in the cell size between strains through statistical analysis as observed *via* TEM (**Figures 3B, C**). Notably, the growth rate of the DKO mutant was reduced as compared to that of strain SC19 (**Figure 3D**). Meanwhile, the CFU counts also showed that strain DKO grew much slower than strain SC19 (**Figure 3E**). Based on these observations, *gidA*-*mnmE* modification pathway appears to be critical for *S. suis* growth, and has no effect on the cell size. Moreover, proteomics also revealed that many DEPs contributed in growth and division were down-regulated too, which were highly consistent with phenotype in growth.

### GidA-MnmE Pathway Contributes to the Pathogenicity of SC19

Experimental infection of mice was performed to estimate the difference of viability *in vivo* between Δ*gidA*Δ*mnmE* and SC19. Firstly, groups of 20 mice were intraperitoneally infected with 3 × 10^9^ CFU/mouse Δ*gidA*Δ*mnmE* and SC19, respectively. Physiological saline was used as a negative control for the other 10 mice. We observed that all mice infected with SC19 that developed severe clinical symptoms, such as septicemia and meningitis died within 1 dpi (10/10). By contrast, only one mouse infected with Δ*gidA*Δ*mnmE* died within 1 dpi, one mouse died within 2 dpi, and another 8 mice were survived during the 7-day observational period (**Figure 2C**). This result indicated that the pathogenicity of Δ*gidA*Δ*mnmE* was remarkably attenuated compared to SC19.

**Figure 4:**
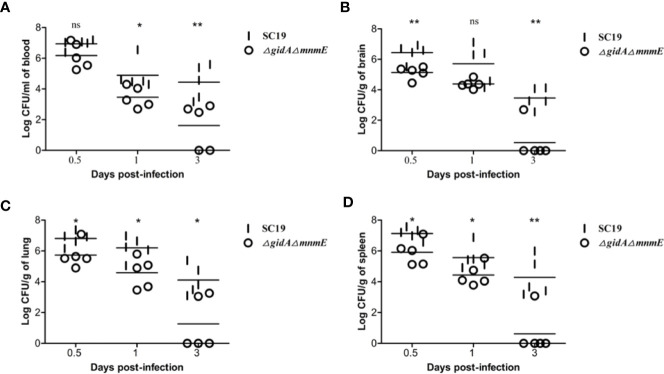
Bacteria loads in different mouse organs. Bacteria loads **(A)** in blood, **(B)** in brain, **(C)** in lung, and **(D)** in spleen. Mice were inoculated intraperitoneally with 1 × 10^8^ CFU of a 1:1 mixture of mid-log phase SC19 and Δ*gidA*Δ*mnmE*. The survival strains were enumerated by plating serial dilutions of the samples on selective plates. Data are the result of CFU/ml or CFU/g in different organs analyzed per sample ± SEM. Statistical significance was determined using the two-tailed *t* test (ns, *p* > 0.05; *, *p* < 0.05; **, *p* < 0.01; ***, *p* < 0.001).

Then, to further evaluate the pathogenicity of Δ*gidA*Δ*mnmE*, a colonization experiment was performed using the intraperitoneal injection with the mixtures of SC19 and Δg*idA*Δ*mnmE* at the ratio of 1:1 (1× 10^8^ CFU/mouse). On account of the growth defect of Δ*gidA*Δ*mnmE in vitro*, we wondered whether the characteristics would be the same under *in vivo*. Sure enough, the *in vivo* adaptability of Δ*gidA*Δ*mnmE* was decreased. The efficiency of colonization of SC19 was much higher at 0.5, 1 and 3 dpi than that of the DKO mutant in blood, brain, lung, and spleen (**Figure 4**). In addition, the DKO mutant strain was almost cleared at the 3 dpi. The results showed that GidA-MnmE pathway contributed to the survival in blood and the colonization in brain, lung, and spleen.

### Targeted Metabolomic Analysis of Amino Acids Obtained From Δ*gidA*Δ*mnmE* and SC19

**Figure 5:**
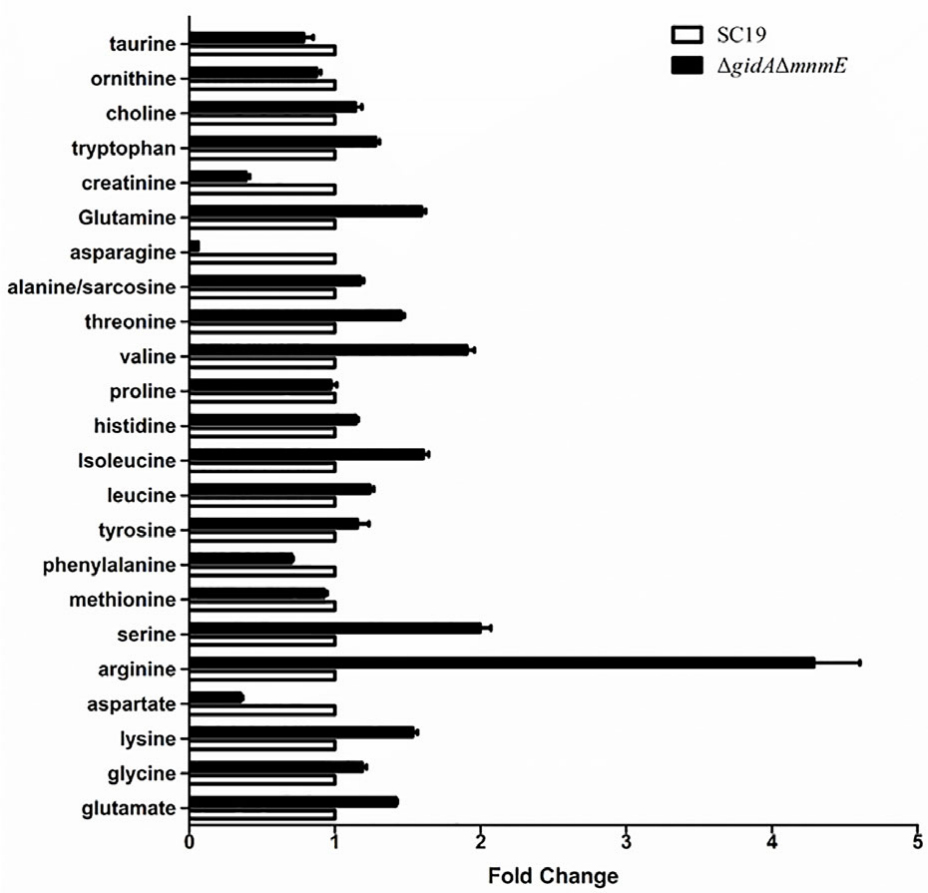
Identified 23 amino acids metabolites between SC19 and Δ*gidA*Δ*mnmE*.

To further study the effects of GidA-MnmE modification pathway on arginine metabolism, we performed targeted metabolomic analysis of amino acids in Δ*gidA*Δ*mnmE* and SC19. Column graph of metabolites analysis showed that in total 23 amino acids metabolites identified by liquid chromatography-tandem mass spectrometry (LC-MS/MS) (**Figure 5**) (RSD of QC samples < 30%). Of the identified amino acids, arginine was significantly increased, while aspartate, asparagine and creatinine were decreased in Δ*gidA*Δ*mnmE* compared to the WT (Fold change > 2 or < 0.5, p < 0.05) (**Table 5**). Up-regulation of arginine in metabolites analysis was in accordance with down-regulation of ADS system in proteomic analysis, indicating that arginine was accumulated in Δ*gidA*Δ*mnmE* owing to blocking the action of GidA-MnmE modification pathway.

**Table 5:** Identified differential amino acid metabolites between the △*gidA*△*mnmE* and SC19.

Amino acid metabolites	QC-RSD	Fold change(Δ*gidA*Δ*mnmE*/SC19)		*p*-value
arginine	0.166065	4.1919		5.76837E-11
aspartate	0.141512	0.3504		9.75737E-10
asparagine	0.062687	0.0606		5.66526E-15
Creatinine\\	0.058136	0.3914		5.91294E-06

## Discussion

GidA and MnmE are evolutionarily conserved tRNA modification enzymes involved in the addition of the cmnm group onto wobble uridine of tRNA, and this modification pathway are crucial for proper and efficient protein translation. Several previous studies have implicated this pathway in connection with pathogenic regulatory mechanism as deletion of single or double gene of *gidA* and *mnmE* has attenuated several bacterial pathogens like S*almonella enterica serovar Typhimurium*, *Pseudomonas syringae*, *Aeromonas hydrophila*, and many others. Despite some progress concerning the contribution of GidA-MnmE modification pathway in pathogenic regulation, the precise mechanism remains elusive. SS is an important zoonotic pathogen, and the role of GidA-MnmE modification pathway in SS is unclear. Here, through “-omics” approaches, we found that SS2 GidA-MnmE pathway can regulate expression of proteins involved in bacterial virulence, and this result was consistent with that of previous study. Moreover, GidA-MnmE pathway affects 15 proteins that are involved in cell division and growth, which is why DKO mutant strain has defect in growth compared to SC19. Particularly, arginine metabolism assay combined with proteomic and metabolomic analysis have revealed that GidA-MnmE modification pathway is a new way in regulation of arginine metabolism of *Streptococcus suis*.

After obtaining the Δ*gidA*Δ*mnmE* mutant strain, we found that the colonies of the mutant appeared smaller than those of SC19 when cultured on TSA plates overnight (**Figure 1A**). So we guess, the DKO mutant may have defect in growth. As expected, both OD600 value and CFU counts showed that the strain DKO grew much slower than strain SC19 (**Figures 1C, D**). In addition, the smaller colonies size was irrelevant to cell size, which was confirmed through TEM observation (**Figure 1B**). To further understand the mechanism behind growth regulation by GidA-MnmE pathway, TMT-based proteomic analysis was conducted. According to the DPE analysis, there were 15 proteins involved in cell division and growth down-regulated in the DKO mutant, such as DnaE, FtsK, StpK, MurD, MurG, and PBP1A, these proteins were all reported to be positively regulated cell division. Our previous study found that StpK is a central regulator that plays an important role in cell growth and division (Zhang et al., 2017). Because phosphorylation level of StpK substrates, such as FtsA, GpsB, DivIVA, and MapZ, that were all crucial cell division-associated proteins are reduced (Grangeasse and Lesterlin; Raaphorst et al., 2017; Sven and Lewis, 2019). Therefore, down-regulation of StpK may defect the cell growth and division of DKO mutant by affecting phosphorylation of these substrates. As far as we know, bacterial cells grow and divide by elongating the lateral cell wall and building a new cell wall disc, then the septum divides the mother cell into two identical daughter cells (Massidda et al., 2013). In this process, a series of enzymes are needed to synthesize new peptidoglycan, involving six MurA-F, PBPs family. These enzymes are attractive drug targets as it is essential and ubiquitous in bacteria but absent in mammalian cells (Azam and Jupudi, 2019). In addition, PBP1A, which catalyzes the transglycosylation and transpeptidation reactions is already the target of β-lactams antibiotics (Ali et al., 2017). On that basis, down-regulation of these growth and division-associated proteins (**Table 4**) is the underlying cause of attenuated growth rate of the Δ*gidA*Δ*mnmE* mutant strain. These findings provide functional context that GidA-MnmE modification pathway is a new regulation method in cell growth and division.

The double knock-out of *gidA* and *mnmE* in SS2 also resulted in an alteration to bacterial pathogenicity. The mouse infection experiment revealed that Δ*gidA*Δ*mnmE* mutant was associated with significantly decreased mortality and bacterial loads. This attenuation may result from the impaired growth of Δ*gidA*Δ*mnmE* or impaired translation efficiency of the virulence-associated proteins, or the dual functions of impaired growth and decreased virulence-associated proteins. SadP and FbpS are adhesin, and HlyC is hemolysin, both of which were down-regulated. Proteins involved in adherence and hemolysis are supposed to be virulence factor, on account of important role in host-bacteria interactions (Segura et al., 2017). In SS, FbpS functions both as an adhesin, promoting bacteria attachment to host cells, and as a bacterial factor, activating signaling pathways (Musyoki et al., 2016). Moreover, in this study, ADS including four DEPs, ArcA, ArcB, and ArcC were down-regulated, only ArgR was up-regulated. ArcABC of SS2 contributes to environmental adaptability, adhesion to and invasion in epithelial cells, as well as resistance to oxygen, nutrient starvation, and acidic environments (Maneerat et al., 2017). ArgR is an essential transcriptional regulator which positively regulates the arcABC operon in mRNA level, however, the protein expression of ArcABC was remarkably down-regulated rather than up-regulated. These results revealed that GidA-MnmE modification pathway has a major impact on ADS expression at translation level, which is far more effective than ArgR as a transcriptional regulator of ADS at transcription level. In other word, the function of ArgR is limited to *arcABC* operon regulation (Willenborg et al., 2016). We also performed a RT-qPCR experiment that examined the mRNA levels of the *arcABC* genes, *arcA* was down-regulated to 2 fold in Δ*gidA*Δ*mnmE* than that in SC19, however, mRNA levels of the *arcB* and *arcC* were unchanged. The result confirmed that ArgR was limited to *arcABC* operon regulation and regulative efficiency was different on *arcA* and *arcBC*, it could be due to different physical distances between *argR* and *arcABC*. Considering all of the above analysis, decreased pathogenicity of Δ*gidA*Δ*mnmE* can be explained by the down-regulation of the above DEPs, which is also consistent to previous reports. In future studies, more attention should be paid to regulation of ADS by GidA-MnmE modification pathway.

**Figure 6:**
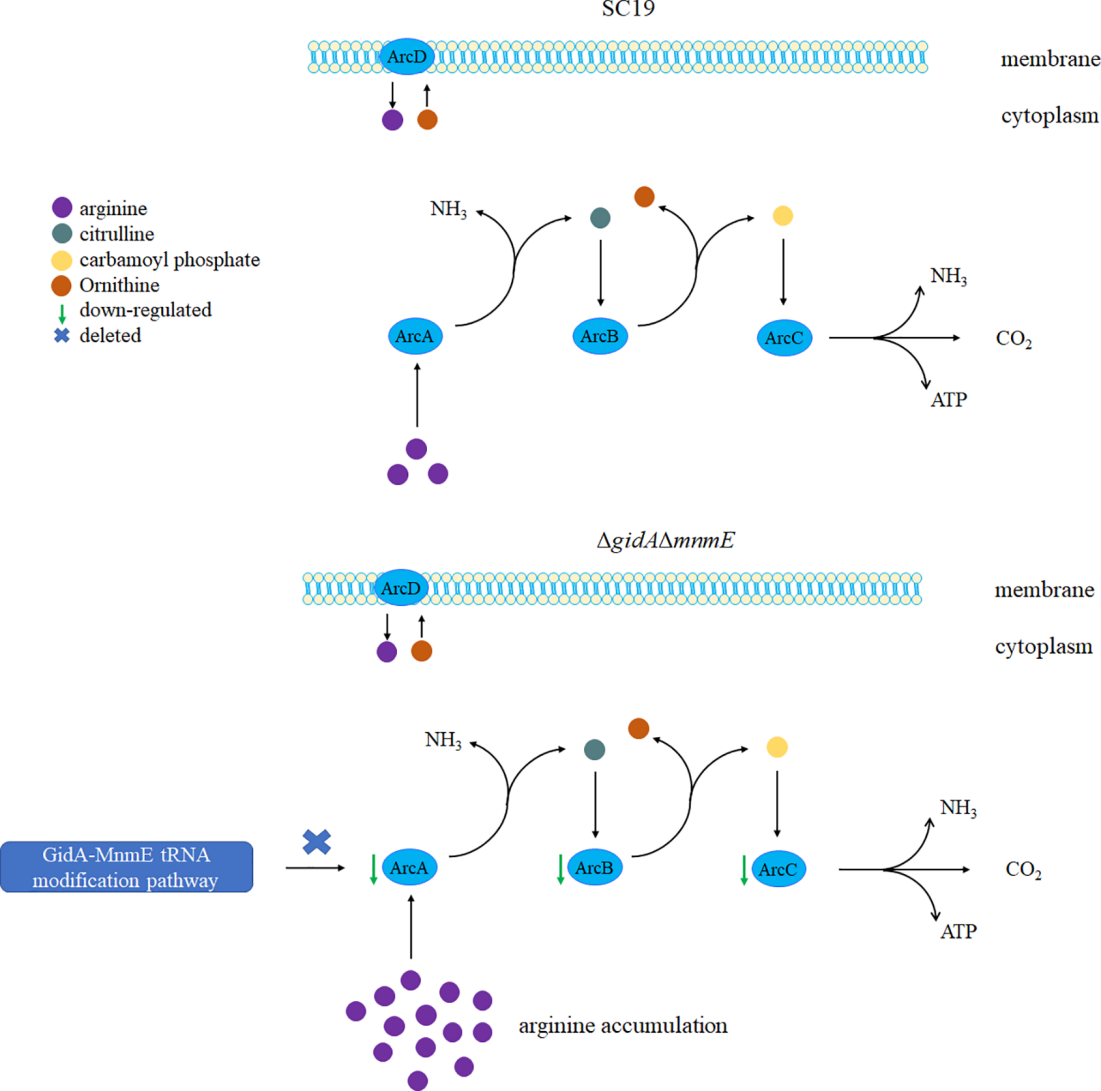
A proposed model for molecular mechanism on arginine metabolism perturbation by GidA-MnmE modification pathway.

According to the GO enrichment analysis, internal relations between DEPs was uncovered. Several biological pathways were significantly perturbed following *gidA* and *mnmE* deletion, such as fatty acid biosynthesis and metabolism, amino acid biosynthesis and metabolism, purine biosynthesis and metabolism (**Table 3**). Here, arginine metabolic process which belong to amino acid biosynthesis and metabolism, is a primary point of our concern. In SS, three core enzymes of ADS are responsible for arginine metabolism as mentioned above. Arginine deiminase (ArcA) catalyzes the conversion of arginine to citrulline; ornithine carbamoyltransferase (ArcB) catalyzes the phosphorolysis of citrulline, yielding ornithine and carbamoyl phosphate; carbamate kinase (ArcC) catalyzes the transfer of phosphate from carbamoyl phosphate to ADP, finally generating ATP, NH_3_, and CO_2_ (Sakanaka et al., 2015; Willenborg et al., 2016). First, we found that AD Activity and ammonia production were decreased in Δ*gidA*Δ*mnmE* mutant, then proteomic analysis was performed, considering the effect on translation efficiency by GidA-MnmE modification pathway. As expected, ArcA, ArcB, and ArcC were down-regulated in the mutant strain, which conformed that blocking this modification pathway could perturb arginine metabolism. In order to comprehensively study the mechanism of GidA-MnmE pathway on regulating arginine metabolism, targeted metabolomics of amino acids was carried out. Moreover, a proposed schematic representation of *S. suis* arginine metabolism perturbed by GidA-MnmE pathway was presented in **Figure 6**. Arginine was significantly upregulated in the Δ*gidA*Δ*mnmE*, the result showed that down-regulation of ArcA, ArcB, and ArcC in the protein profile led to arginine accumulation in metabolic level in the Δ*gidA*Δ*mnmE*, which can explain the differences in proteomic phenotypes between SC19 and Δ*gidA*Δ*mnmE*. All of these reflected the conduction from upstream to downstream by GidA-MnmE modification pathway.

In summary, our *in vitro* and *in vivo* studies combined with proteomic and metabolomic experiments clearly demonstrate the important roles of GidA-MnmE modification pathway on the growth, pathogenicity, and particularly on arginine metabolism of SS2. In addition, arginine was shown as a potential biomarker for perturbation of GidA-MnmE modification pathway. These findings provide new insights to better understanding the modification function of GidA-MnmE modification pathway, and highlight the important link between GidA-MnmE pathway and arginine metabolism.

## Data Availability Statement

The datasets presented in this study can be found in online repositories. The names of the repository/repositories and accession number(s) can be found in the article/**Supplementary Material**.

## Ethics Statement

The animal study protocol was approved by the Ethics Committee of Institute of Animal Husbandry and Veterinary, Hubei Academy of Agricultural Sciences (Wuhan, China).

## Author Contributions

TG conceived and designed this project and experiments. TG, ZL, WeL and WaL performed the experiments. DZ, KY, GZ, and RG analyzed the data. TG, FY, and YT contributed to the development of the figures and tables. TG, RZ, FY, and YT wrote the manuscript. All authors reviewed the manuscript. All authors contributed to the article and approved the submitted version.

## Funding

This work was supported by the National Key R&D Program of China (2017YFD0500201), the Natural Science Foundation of China (NSFC; Grant No. 31802189, 31672560), the Natural Science Foundation of Hubei Province (2018CFA045), Hubei Province Innovation Center of Agricultural Sciences and Technology (2019-620-000-001-017), Key Laboratory of Prevention and Control Agents for Animal Bacteriosis (Ministry of Agriculture) (KLPCAAB-YTP-1802), and Science Foundation of Hubei Academy of Agricultural Science (2018NKYJJ11).

## Conflict of Interest

The authors declare that the research was conducted in the absence of any commercial or financial relationships that could be construed as a potential conflict of interest.

## Supplementary Material

Supplementary Figure 1
**(A)** PCR confirmation of the Δ*gidA*Δ*mnmE* mutant. Lanes 1 to 3 represent the amplification of the *gidA* gene using the primer set *gidA*-F/*gidA*-R. Lanes 4 to 6 represent the amplification of the *mnmE* gene using the primer set *mnmE* -F/*mnmE* -R. Lanes 7 to 9 represent the amplification of the *cps2J* gene using the primer set *cps2J* -F/*cps2J* -R. Lanes 1, 4, and 7 use DNA of SC19 as templates, whereas Lanes 2, 5, and 8 use DNA of Δ*gidA*Δ*mnmE* as templates, Lanes 3, 6, and 9 was negative control. **(B)** Confirmation of the Δ*gidA*Δ*mnmE* mutant by RT-PCR. Lanes 1 to 3 represent the amplification of the *gidA* gene using the primer set *gidA*-F/*gidA*-R. Lanes 4 to 6 represent the amplification of the *mnmE* gene using the primer set *mnmE* -F/*mnmE* -R. Lanes 7 to 9 represent the amplification of the *cps2J* gene using the primer set *cps2J* -F/*cps2J* -R. Lanes 1, 4, and 7 use cDNA of SC19 as templates, whereas Lanes 2, 5, and 8 use cDNA of Δ*gidA*Δ*mnmE* as templates, Lanes 3, 6, and 9 was negative control.

Supplementary Figure 2 Gene expression analysis of *arcABC* genes of Δ*gidA*Δ*mnmE* compared with SC19.
