# Supplementary figures and images for: Proteomic and Metabolomic Analyses Provide Insights into the Mechanism on Arginine Metabolism Regulated by tRNA Modification Enzymes GidA and MnmE of Streptococcus suis
Source: Front Cell Infect Microbiol. 2020 Dec 11;10:597408. doi: 10.3389/fcimb.2020.597408 (PMC7793837; doi:10.3389/fcimb.2020.597408)

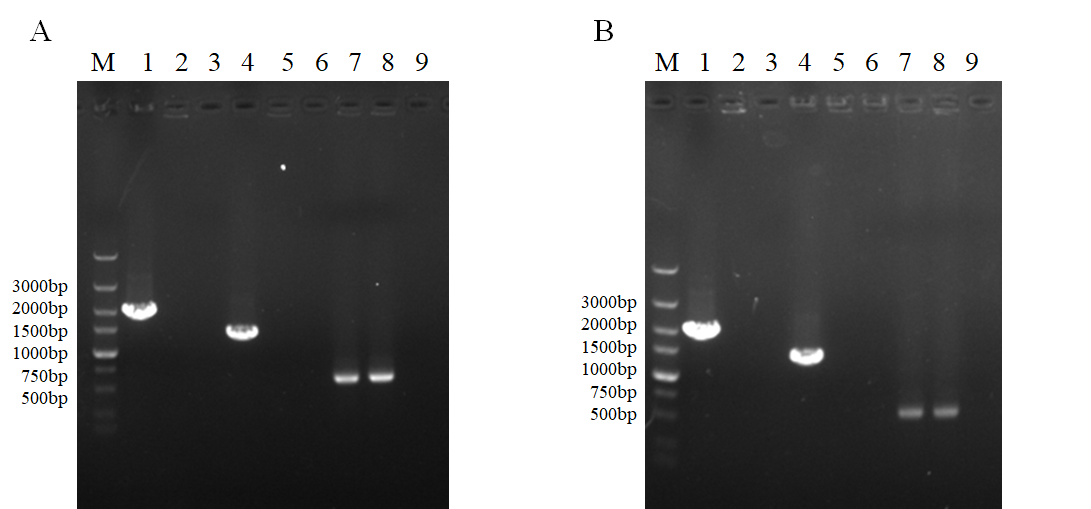

Supplement: Supplementary Figure 1 — (A) PCR confirmation of the ΔgidAΔmnmE mutant. Lanes 1 to 3 represent the amplification of the gidA gene using the primer set gidA-F/gidA-R. Lanes 4 to 6 represent the amplification of the mnmE gene using the primer set mnmE -F/mnmE -R. Lanes 7 to 9 represent the amplification of the cps2J gene using the primer set cps2J -F/cps2J -R. Lanes 1, 4, and 7 use DNA of SC19 as templates, whereas Lanes 2, 5, and 8 use DNA of ΔgidAΔmnmE as templates, Lanes 3, 6, and 9 was negative control. (B) Confirmation of the ΔgidAΔmnmE mutant by RT-PCR. Lanes 1 to 3 represent the amplification of the gidA gene using the primer set gidA-F/gidA-R. Lanes 4 to 6 represent the amplification of the mnmE gene using the primer set mnmE -F/mnmE -R. Lanes 7 to 9 represent the amplification of the cps2J gene using the primer set cps2J -F/cps2J -R. Lanes 1, 4, and 7 use cDNA of SC19 as templates, whereas Lanes 2, 5, and 8 use cDNA of ΔgidAΔmnmE as templates, Lanes 3, 6, and 9 was negative control. [file Image_1.tif]
